# Supplementary material for: Extracellular Vesicles and Their miRNA Content in Amniotic and Tracheal Fluids of Fetuses with Severe Congenital Diaphragmatic Hernia Undergoing Fetal Intervention
Source: Cells. 2021 Jun 14;10(6):1493. doi: 10.3390/cells10061493 (PMC8231823; doi:10.3390/cells10061493)
Supplement: Supplementary file 1 [file cells-10-01493-s001.zip › cells-1255303 sm pdf/S1_table.pdf]

Supplementary Table S1

MISEV 2018 guidelines compliance

	**Section title**	**Required information according to MISEV2018**	**Mandatory requirement**	**Not applicable/not available**	**Our approach**	**Compliance with MISEV2018 requirements**
1	Nomenclature	The term extracellular vesicle (EV) can be used with demonstration of extracellular (no intact cells) and vesicular nature per these characterization and function	YES		The term extracellular vesicle (EV) has been used in the manuscript	YES
2a	Collection and pre-processing (tissue culture conditioned medium)	General cell characterization	YES	N/A		
2a	Collection and pre-processing (tissue culture conditioned medium)	Medium used before and during collection (additives, serum, other)	YES	N/A		
2a	Collection and pre-processing	Exact protocol for depletion of EVs from	YES	N/A		

	(tissue culture conditioned medium)	additives in collection medium				
2a	Collection and pre-processing (tissue culture conditioned medium)	Nature and size of culture vessels, and volume of medium during conditioning<br>e) specific culture conditions (treatment, % O2, coating,polarization…) before and during collection	N/A			
2a	Collection and pre-processing (tissue culture conditioned medium)	Number of cells/ml and % of live/ dead cells at time of collection	N/A			
2a	Collection and pre-processing (tissue culture conditioned medium)	Frequency and interval of Conditioned Medium harvest	N/A			
2b and 2c	Collection and pre-processing (Biofluids or	Donor status if available (age, sex, food/water	YES		Complete clinical information on maternal status were	YES

	tissues)	intake, collection time, disease, medication, other)			available.	
2b and 2c	Collection and pre-processing (Biofluids or tissues)	Volume of biofluid or volume/mass of tissue sample collected per donor	YES		For each subject, we recorded the total volume of amniotic/tracheal fluid collected	YES
2b and 2c	Collection and pre-processing (Biofluids or tissues)	Total volume/mass used for EV isolation (if pooled from several donors)	YES		EVs isolation was performed starting from a standardized volume of fluids for each subject. Each EVs pellet aliquot obtained from each subject was then resuspended in 1 mL of PBS triple filtered (pore size 0.1 µm).	YES
2b and 2c	Collection and pre-processing (Biofluids or tissues)	All known collection conditions, including additives, at time of collection			No additives.	YES
2b and 2c	Collection and pre-processing (Biofluids or tissues)	Pre-treatment to separate major fluid-specific contaminants before EV isolation			Samples (amniotic and tracheal fluids) were centrifuged three times at increasing speeds (1000 g, 2000 g, 3000 g) for 15 min at 4 °C to remove cell debris and aggregates.	YES

2b and 2c	Collection and pre-processing (Biofluids or tissues)	Temperature and time of biofluid/tissue handling before and during pre-treatment			Each sample was processed within 2 hrs from collection foe EV characterization. An EV pellet was frozen at -80°C for miRNA extraction.	YES
2b and 2c	Collection and pre-processing (Biofluids or tissues)	For cultured tissue explants: volume, nature of medium and time of culture before collecting conditioned medium		N/A		
2b and 2c	Collection and pre-processing (Biofluids or tissues)	For direct tissue EV extraction: treatment of tissue to release vesicles without disrupting cells		N/A		
2d	Storage and recovery	Storage and recovery (e.g., thawing) of CCM, biofluid, or tissue before EV isolation (storage temperature, vessel, time; method of thawing or other sample preparation)	N/A			

2d	Storage and recovery	Storage and recovery of EVs after isolation (temperature, vessel, time, additive(s)…)	YES		Isolated EVs were immediately characterized by flow cytometry and Nanosight. . An EV pellet was frozen at -80°C for miRNA extraction.	YES
3	EV separation and concentration	Centrifugation: reference number of tube(s), rotor(s), adjusted k factor(s) of each centrifugation step (= time+ speed+ rotor, volume/density of centrifugation conditions), temperature, brake settings	YES		We applied a differential ultracentrifugation with previous lower-speed steps. Amniotic and tracheal fluids were centrifuged at 1000, 2000, and 3000 × g for 15 min at 4 °C (Haereus Labofuge 400R, Hanau, Germany). The obtained pellets were discarded to remove cells, apoptotic bodies and cell debris. EVs were then isolated from supernatants by ultracentrifugation at 110,000 × g for 75 minutes at 4 °C in polypropylene ultracentrifuge tubes (Quick-Seal ultra-clear centrifuge tubes, Beckman Coulter; Brea, CA, USA) rotor MLA-55 (Beckman Coulter), filled with PBS previously filtered through a 0.10-µm pore-size polyethersulfone filter (StericupRVP, Merck Millipore; Burlington, MA,	YES

					USA). Our method is included in the category “Intermediate recovery, intermediate specificity = mixed EVs with limited non-EV components”	
3	EV separation and concentration	Density gradient: nature of matrix, method of generating gradient, reference (and size) of tubes, centrifugation speed and time (with brake specified), method and volume of fraction recovery.		N/A		
3	EV separation and concentration	Chromatography: matrix (nature, pore size,…), loaded sample volume, fraction volume, number		N/A		
3	EV separation and concentration	Precipitation: reference of polymer, ratio vol/vol or weight/vol polymer/fluid, time/temperature of incubation, time/speed/temperatur		N/A		

		e of centrifugation				
3	EV separation and concentration	Filtration: reference of filter type (=nature of membrane, pore size…), time and speed of centrifugation, volume before/after (in case of concentration)		N/A		
3	EV separation and concentration	Antibody-based : reference of antibodies, mass Ab/amount of EVs, nature of Ab carrier (bead, surface) and amount of Ab/carrier surface		N/A		
3	EV separation and concentration	Other…: all necessary details to allow replication		N/A		
3	EV separation and concentration	Additional step(s) to concentrate, if any		N/A		
3	EV separation and concentration	Additional step(s) to wash matrix and/or sample, if any		N/A		
4a	EV characterization,	Volume of fluid, and/or cell number, and/or tissue mass used to	N/A			

	Quantification	isolate EVs				
4a	EV characterization, Quantification	Global quantification by at least 2 methods: protein amount, particle number, lipid amount, expressed per volume of initial fluid or number of producing cells/mass of tissue	YES		In order to quantify the total number of EVs we applied the two following approaches:<br><br>1) Nanoparticle tracking analysis by NanoSight LM10-HS system (Malvern Panalytical Ltd, Malvern, UK). Five 30-s recordings were made for each sample. Collected data were analyzed with NTA software (Malvern Panalytical Ltd.), which provided high-resolution particle-size distribution profiles as well as measurements of the EV concentration.<br><br>2) High resolution Flow cytometry by MACSQuant, Miltenyi Biotec. In order to analyze EV integrity, 60 µl aliquots were stained with 0.2 µM 5(6)-carboxyfluorescein diacetate N-succinimidyl ester (CFSE) at 37 °C for 20 min in the dark. CFSE is a cell-permeant, non-fluorescent pro-dye. If incorporated into	YES

					intact EVs, which contain esterases as live cells, the acetate groups of CFSE is cleaved producing a membrane-impermeant molecule with green fluorescence.	
4a	EV characterization, Quantification	Ratio of the 2 quantification figures	YES	N/A		
4b	EV characterization, General Characterization	At least three positive protein markers of EVs, including at least one transmembrane/lipid bound protein and one cytosolic protein<br><br>At least one negative protein marker	YES		The following antibodies have been used to measure positive protein markers of plasmatic EVs:<br><br>CD66+ (neutrophils)<br>CD14+ (monocytes/macrophages)<br>CD105+ (endothelium)<br>EpCAM+ (epithelium)<br>HLAG+ (Trophoblast)<br>HERV-W+ (Syncytin-1 protein, placenta)<br><br>Transmembrane, tissue specific and focus of the present study.<br><br>CFSE staining was used to assess cytosolic esterase	YES

					activity. As negative control, representative EVs samples were incubated with Tween20 (P1379; Merk, Sigma-Aldrich) ON at 37 °C, and then incubated with CFSE as described above. Positivity for CFSE fluorescence was evaluated by High Resolution Flow cytometry analysis	
4c	Single EV characterization	Images of single EVs by electron microscopy	YES		Transmission Electron Microscopy (TEM) analysis was performed on random samples as quality control.	YES
4c	Single EV characterization	Non-image-based method analysing large numbers of single EVs: Non-image-based method analysing large numbers of single EVs: NTA, TRPS, FCS, high-resolution flow cytometry, multi-angle light-scattering, Raman spectroscopy, etc.	YES		NTA and Flow Cytometry were performed	YES
5	Functional studies	Dose-response assessment	N/A			

5	Functional studies	Negative control = nonconditioned medium, biofluid/tissue from control donors, as applicable	N/A		.	
5	Functional studies	Quantitative comparison of functional activity of total fluid, vs EV-depleted fluid, vs EVs (after high recovery/low specificity separation)	N/A			
5	Functional studies	Quantitative comparison of functional activity of EVs vs other EPs/fractions after low recovery/high specificity separation	N/A			
5	Functional studies	Quantitative comparison of activity of EV subtypes (if subtype-specific	N/A			

		function claimed)				
5	Functional studies	Extent of functional activity in the absence of contact between EV donor and EV recipient	N/A			
6	Reporting	Submission of data (proteomic, sequencing, other) to relevant public, curated databases or open-access repository	YES	N/A		
