# Supplementary material for: Extracellular Vesicles and Their miRNA Content in Amniotic and Tracheal Fluids of Fetuses with Severe Congenital Diaphragmatic Hernia Undergoing Fetal Intervention
Source: Cells. 2021 Jun 14;10(6):1493. doi: 10.3390/cells10061493 (PMC8231823; doi:10.3390/cells10061493)
Supplement: Supplementary file 1 [file cells-10-01493-s001.zip › cells-1255303 sm pdf/S2_table.pdf]

Table S2. Mean concentrations of EV subtypes in survivors and non-survivors estimated by linear Poisson regression models, allowing for overdispersion.

	Tracheal fluid					Amniotic fluid				
	Survivors		Non-survivors		P-value	Survivors		Non-survivors		P-value
	Mean	95% CI	Mean	95% CI		Mean	95% CI	Mean	95% CI	
**Nanoparticle Tracking Analysis** (count *10^6/ml)										
**Total EV**	384307	(383811;384804)	1197863	(1196625;1199102)	**<0,0001**	19936	(19812;20060)	25573	(25112;25390)	**<0,0001**
**Exosomes**	16888	(16784;16992)	32847	(32643;33053)	**<0,0001**	304	(289;319)	322	(307;338)	0,0965
**MV**	367420	(366935;367905)	1165016	(1163795;1166238)	**<0,0001**	19632	(19510;19755)	25251	(25112;25390)	**<0,0001**
**Flow Cytometry** (count *10^3/ml)										
**CD66+ (neutrophils)**	89	(81;97)	86	(76;97)	0,6873	344	(326;362)	486	(467;506)	**<0,0001**
**CD14+ (monocytes)**	57	(51;63)	76	(67;87)	**0,0005**	264	(249;281)	376	(360;394)	**<0,0001**
**CD105+ (endothelium)**	52	(47;58)	78	(69;89)	**<0,0001**			n/a		
**EpCAM+ (epithelium)**	60	(54;66)	79	(69;89)	**0,0010**	251	(236;267)	390	(373;407)	**<0,0001**
**HLAG+ (placenta)**			n/a			272	(256;288)	389	(372;407)	**<0,0001**
**HERV-W+ (placenta)**			n/a			290	(273;307)	329	(314;345)	**0,0008**
