# Supplementary material for: Extracellular Vesicles and Their miRNA Content in Amniotic and Tracheal Fluids of Fetuses with Severe Congenital Diaphragmatic Hernia Undergoing Fetal Intervention
Source: Cells. 2021 Jun 14;10(6):1493. doi: 10.3390/cells10061493 (PMC8231823; doi:10.3390/cells10061493)
Supplement: Supplementary file 1 [file cells-10-01493-s001.zip › cells-1255303 sm pdf/S3_table.pdf]

Table S3. Screening of miRNA expression levels. Mean concentrations of EV-miRNA levels in survivors and non-survivors measured by OpenArray.

			Amniotic Fluids					Tracheal Fluids				
**miRBase ID (v21)**	**miRNA name**	**Quant Studio Assay ID**	**Fold Chang e**	**Mean non-survivors**	**Mean survivors**	**P-value**	**Chosen for validation**	**Fold Change**	**Mean non-survivors**	**Mean survivors**	**P-value**	**Chosen for validation**
hsa-let-7a-5p	let_7a_000377	000377	1.353	242.016	178.841	0.4230		1.597	612.076	383.159	0.1331	
hsa-let-7b-5p	let_7b_002619	002619	0.004	0.201	49.518	0.3071		Not expressed				
hsa-let-7c-5p	let_7c_000379	000379	1.392	47.177	33.890	0.2826		1.256	97.361	77.546	0.2174	
hsa-let-7d-3p	let_7d__001178	001178	0.807	0.864	1.071	0.5205		1.506	7.739	5.138	0.1807	
hsa-let-7d-5p	let_7d_002283	002283	1.098	54.653	49.775	0.7642		1.285	156.696	121.983	0.8549	
hsa-let-7e-3p	let_7e__002407	002407	0.741	0.201	0.271	0.2907		0.899	0.393	0.437	0.2164	
hsa-let-7e-5p	let_7e_002406	002406	1.499	487.686	325.234	0.0987		1.219	1188.591	975.204	0.5560	
hsa-let-7f-2-3p	let_7f_2__002418	002418	Not expressed					0.457	0.186	0.406	0.0560	
hsa-let-7f-5p	let_7f_000382	000382	1.956	55.095	28.162	0.1673		1.819	133.463	73.353	0.0891	
hsa-let-7g-5p	let_7g_002282	002282	1.258	69.266	55.042	0.7924		1.851	220.327	119.010	0.0996	
hsa-miR-100-5p	miR_100_000437	000437	1.490	998.499	670.145	0.2537		1.992	1131.621	568.191	0.1279	
hsa-miR-101-3p	miR_101_002253	002253	1.761	3.102	1.761	0.0761		2.398	10.161	4.237	0.2013	
hsa-miR-103a-3p	miR_103_000439	000439	1.377	106.336	77.200	0.3344		1.744	261.732	150.060	0.2244	
hsa-miR-106a-5p	miR_106a_002169	002169	1.081	1593.403	1473.507	0.6935		1.482	6576.707	4438.804	0.2760	
hsa-miR-106b-3p	miR_106b__00238 0	002380	Not expressed					4.426	25.333	5.723	0.0918	
hsa-miR-106b-5p	miR_106b_000442	000442	1.423	235.900	165.809	0.3463		1.812	928.627	512.599	0.7374	
hsa-miR-107	miR_107_000443	000443	1.621	4.016	2.477	0.0882		1.819	14.901	8.191	0.2185	
hsa-miR-10a-3p	miR_10a__002288	002288	1.047	0.254	0.243	0.4337		Not expressed				
hsa-miR-10a-5p	miR_10a_000387	000387	1.186	218.433	184.197	0.6094		0.362	6.825	18.869	0.6966	
hsa-miR-10b-3p	miR_10b__002315	002315	0.972	40.183	41.324	0.9099		0.403	1.048	2.597	0.8845	
hsa-miR-10b-5p	miR_10b_002218	002218	0.932	105.442	113.149	0.8405		0.264	1.778	6.733	0.2536	
hsa-miR-1180-3p	miR_1180_002847	002847	0.880	17.049	19.370	0.9897		1.057	43.419	41.070	0.2423	
hsa-miR-1183	miR_1183_002841	002841	1.078	0.347	0.322	0.8197		Not expressed				
hsa-miR-122-5p	miR_122_002245	002245	99.636	96.221	0.966	0.7778		2.303	0.765	0.332	0.0397	
hsa-miR-1226-5p	miR_1226__00275	002758	0.974	1.074	1.103	0.9917		0.155	0.186	1.200	0.1608	

	8									
hsa-miR-1227-3p	miR_1227_002769	002769	4.666	10.814	2.318	0.3157	4.394	5.874	1.337	0.6009
hsa-miR-1233-3p	miR_1233_002768	002768	0.036	0.201	5.565	0.2870	0.279	0.186	0.665	0.2395
hsa-miR-124-3p	miR_124a_001182	001182	0.724	0.201	0.278	0.0916	Not expressed			
hsa-miR-1244	miR_1244_002791	002791	1.019	1.039	1.020	0.8765	1.090	0.858	0.788	0.5217
hsa-miR-1247-5p	miR_1247_002893	002893	2.800	214.098	76.457	0.1682	0.172	0.186	1.081	0.1719
hsa-miR-1255b-5p	miR_1255B_002801	002801	3.018	1.831	0.607	0.1444	3.556	3.078	0.865	0.1713
hsa-miR-125a-3p	miR_125a_3p_002199	002199	1.104	0.268	0.243	0.9674	0.503	0.364	0.724	0.0560
hsa-miR-125a-5p	miR_125a_5p_002198	002198	0.516	18.934	36.707	0.1204	1.025	98.207	95.772	0.4980
hsa-miR-125b-1-3p	miR_125b_1__002378	002378	1.955	0.475	0.243	0.2959	1.498	0.559	0.373	0.5606
hsa-miR-125b-2-3p	miR_125b_2__002158	002158	Not expressed				0.027	0.186	6.755	0.0278
hsa-miR-125b-5p	miR_125b_000449	000449	1.330	675.688	508.199	0.2955	1.827	1345.400	736.302	0.1945
hsa-miR-1260a	miR_1260_002896	002896	1.693	265.512	156.849	0.8105	1.368	167.690	122.543	0.7730
hsa-miR-126-3p	miR_126_002228	002228	1.453	14.838	10.211	0.8878	2.989	34.205	11.443	0.1331
hsa-miR-126-5p	miR_126__000451	000451	1.125	3.096	2.752	0.3434	2.629	4.187	1.593	0.7581
hsa-miR-1267	miR_1267_002885	002885	Not expressed				0.294	0.186	0.632	0.1376
hsa-miR-1269a	miR_1269_002789	002789	1.074	0.261	0.243	0.9178	0.875	0.291	0.332	0.0582
hsa-miR-1270	miR_1270_002807	002807	Not expressed				0.469	0.186	0.396	0.4464
hsa-miR-1271-5p	miR_1271_002779	002779	Not expressed				2.525	1.685	0.667	0.1142
hsa-miR-127-3p	miR_127_000452	000452	2.331	444.504	190.653	0.2961	1.065	11.564	10.859	0.0826
hsa-miR-128-3p	miR_128a_002216	002216	1.143	2.559	2.239	0.5385	1.681	8.540	5.079	0.1619
hsa-miR-1290	miR_1290_002863	002863	1.474	37.040	25.126	0.2677	5.139	88.562	17.232	0.3508
hsa-miR-1291	miR_1291_002838	002838	1.000	1.472	1.472	0.8942	1.286	1.194	0.928	0.8457
hsa-miR-1303	miR_1303_002792	002792	3.103	2.514	0.810	0.9621	3.704	5.692	1.537	0.9201
hsa-miR-130a-3p	miR_130a_000454	000454	1.282	501.867	391.588	0.3966	2.254	2843.845	1261.922	0.1619
hsa-miR-130b-3p	miR_130b_000456	000456	1.890	45.936	24.306	0.0926	1.802	47.963	26.610	0.5435
hsa-miR-130b-5p	miR_130b__002114	002114	Not expressed				0.435	0.385	0.883	0.2270
hsa-miR-132-3p	miR_132_000457	000457	1.374	88.321	64.260	0.3891	1.647	129.743	78.783	0.2684
hsa-miR-133a-3p	miR_133a_002246	002246	4.330	8.066	1.863	0.3283	0.253	0.186	0.734	0.1635
hsa-miR-134-5p	miR_134_001186	001186	1.645	1.922	1.169	0.9698	Not expressed			

hsa-miR-135a-5p	miR_135a_000460	000460	1.718	11.895	6.925	0.1421	2.732	11.525	4.218	0.0655
hsa-miR-135b-3p	miR_135b__002159	002159	0.285	0.201	0.705	0.2316	0.141	0.186	1.315	0.0885
hsa-miR-135b-5p	miR_135b_002261	002261	1.533	87.705	57.227	0.2795	1.871	130.686	69.841	0.1459
hsa-miR-136-3p	miR_136__002100	002100	1.748	19.486	11.149	0.1441	0.774	0.563	0.727	0.2151
hsa-miR-137	miR_137_001129	001129	0.956	0.232	0.243	0.8250	1.701	0.565	0.332	0.2115
hsa-miR-138-5p	miR_138_002284	002284	1.124	53.942	48.005	0.9677	1.564	512.136	327.536	0.1337
hsa-miR-140-3p	miR_140_3p_002234	002234	1.186	1.157	0.975	0.7756	1.753	3.802	2.169	0.3235
hsa-miR-140-5p	miR_140_001187	001187	1.043	15.623	14.974	0.8629	1.477	75.795	51.317	0.3343
hsa-miR-141-3p	miR_141_000463	000463	1.603	661.160	412.385	0.7152	2.564	2339.284	912.307	0.1361
hsa-miR-141-5p	miR_141__002145	002145	1.249	0.303	0.243	0.1706	0.507	0.231	0.455	0.1189
hsa-miR-142-3p	miR_142_3p_000464	000464	2.841	4.818	1.696	0.2748	2.399	27.099	11.295	0.0384
hsa-miR-143-3p	miR_143_002249	002249	2.111	2.090	0.990	0.2611	1.546	0.590	0.381	0.9677
hsa-miR-145-5p	miR_145_002278	002278	0.573	0.201	0.351	0.1945	Not expressed			
hsa-miR-146a-5p	miR_146a_000468	000468	1.386	183.780	132.641	0.2683	2.180	518.940	237.997	0.0969
hsa-miR-146b-3p	miR_146b_3p_002361	002361	1.348	0.551	0.408	0.4896	1.840	2.237	1.215	0.0789
hsa-miR-146b-5p	miR_146b_001097	001097	1.174	151.818	129.337	0.5204	2.064	760.272	368.261	0.1795
hsa-miR-147a	miR_147_000469	000469	0.634	0.201	0.318	0.0877	Not expressed			
hsa-miR-148a-3p	miR_148a_000470	000470	1.408	88.308	62.725	0.3988	2.040	199.566	97.845	0.1751
hsa-miR-148b-3p	miR_148b_000471	000471	0.791	2.699	3.412	0.3072	1.024	6.384	6.233	0.5939
hsa-miR-148b-5p	miR_148b__002160	002160	1.410	0.564	0.400	0.7069	1.631	2.602	1.595	0.8018
hsa-miR-149-5p	miR_149_002255	002255	1.193	567.106	475.274	0.4170	0.707	609.664	862.099	0.6375
hsa-miR-150-5p	miR_150_000473	000473	1.046	58.635	56.041	0.5165	0.632	34.958	55.309	0.4268
hsa-miR-151a-3p	miR_151_3p_002254	002254	0.881	37.511	42.560	0.9370	18.929	5791.676	305.974	0.1543
hsa-miR-151a-5p	miR_151_5P_002642	002642	0.922	4.510	4.891	0.5330	1.146	16.549	14.438	0.7793
hsa-miR-152-3p	miR_152_000475	000475	1.579	43.090	27.298	0.1681	1.598	32.754	20.500	0.2904
hsa-miR-154-3p	miR_154__000478	000478	2.874	2.719	0.946	0.3732	0.703	0.234	0.332	0.6843
hsa-miR-154-5p	miR_154_000477	000477	3.679	0.894	0.243	0.2370	Not expressed			
hsa-miR-155-5p	miR_155_002623	002623	2.116	1.721	0.814	0.3674	0.558	3.078	5.519	0.9815
hsa-miR-15a-3p	miR_15a__002419	002419	Not expressed				0.470	0.186	0.395	0.1552
hsa-miR-15a-5p	miR_15a_000389	000389	1.062	4.671	4.397	0.6250	2.287	27.913	12.205	0.1454

hsa-miR-15b-3p	miR_15b__002173	002173	Not expressed					0.017	0.186	11.211	0.2098	
hsa-miR-15b-5p	miR_15b_000390	000390	1.329	110.318	83.033	0.6173		1.967	635.849	323.308	0.0428	
hsa-miR-16-1-3p	miR_16_1__002420	002420	1.349	0.328	0.243	0.6630		1.204	0.598	0.497	0.7536	
hsa-miR-16-5p	miR_16_000391	000391	1.106	521.713	471.523	0.6507		1.580	2954.325	1869.606	0.1593	
**hsa-miR-17-3p**	**miR_17__002421**	**002421**	Not expressed					**0.268**	**0.186**	**0.692**	**0.0084**	*****
hsa-miR-17-5p	miR_17_002308	002308	1.090	1579.202	1448.433	0.4825		1.434	5898.723	4112.506	0.2915	
hsa-miR-181a-2-3p	miR_181a_2__002317	002317	0.891	35.031	39.328	0.8826		1.349	170.307	126.260	0.2789	
hsa-miR-181a-3p	miR_213_000516	000516	1.624	3.778	2.326	0.2637		1.514	24.065	15.898	0.3414	
hsa-miR-181a-5p	miR_181a_000480	000480	1.224	377.265	308.128	0.5150		1.913	1560.126	815.588	0.6047	
hsa-miR-181c-3p	miR_181c__002333	002333	1.725	0.419	0.243	0.3930		2.137	19.160	8.968	0.8812	
hsa-miR-181c-5p	miR_181c_000482	000482	1.433	7.179	5.009	0.7919		1.292	23.726	18.360	0.4710	
hsa-miR-182-5p	miR_182_002334	002334	1.153	0.280	0.243	0.4272		0.565	0.236	0.417	0.1679	
hsa-miR-183-3p	miR_183__002270	002270	0.837	5.091	6.084	0.1115		1.037	9.254	8.921	0.0466	
hsa-miR-183-5p	miR_183_002269	002269	0.263	0.344	1.311	0.4655		0.115	0.186	1.611	0.3855	
hsa-miR-184	miR_184_000485	000485	1.356	26.704	19.696	0.3637		0.122	1.990	16.318	0.2733	
hsa-miR-185-5p	miR_185_002271	002271	0.894	1.114	1.246	0.7817		1.161	3.619	3.117	0.6141	
hsa-miR-186-5p	miR_186_002285	002285	1.059	110.876	104.671	0.7305		1.406	400.081	284.556	0.4208	
hsa-miR-187-3p	miR_187_001193	001193	1.666	0.755	0.454	0.9301		Not expressed				
hsa-miR-188-3p	miR_188_3p_002106	002106	0.593	0.201	0.339	0.1935		Not expressed				
hsa-miR-18a-3p	miR_18a__002423	002423	Not expressed					0.670	0.361	0.540	0.1381	
hsa-miR-18a-5p	miR_18a_002422	002422	1.105	6.335	5.733	0.7504		1.480	24.471	16.536	0.2622	
**hsa-miR-190a-5p**	**miR_190_000489**	000489	**2.546**	**3.912**	**1.537**	**0.0478**	*****	1.728	3.454	1.999	0.1021	
hsa-miR-190b	miR_190b_002263	002263	0.891	14.090	15.807	0.9577		1.226	52.446	42.789	0.4136	
hsa-miR-191-5p	miR_191_002299	002299	1.164	1303.869	1120.564	0.4887		1.144	2762.656	2414.468	0.4440	
hsa-miR-192-3p	miR_192__002272	002272	5.989	1.455	0.243	0.0837		Not expressed				
hsa-miR-192-5p	miR_192_000491	000491	20.496	827.018	40.350	0.4651		3.005	54.801	18.240	0.0856	
hsa-miR-193a-5p	miR_193a_5p_002281	002281	1.668	14.025	8.407	0.1287		0.842	7.309	8.684	0.4792	
hsa-miR-193b-3p	miR_193b_002367	002367	1.772	3598.287	2031.141	0.6937		1.562	685.967	439.096	0.2751	
hsa-miR-193b-5p	miR_193b__002366	002366	0.918	1.269	1.382	0.0908		Not expressed				
hsa-miR-194-5p	miR_194_000493	000493	29.885	640.668	21.438	0.0736		3.175	44.156	13.909	0.1116	

hsa-miR-195-5p	miR_195_000494	000494	1.029	9.260	8.999	0.8289	1.629	44.309	27.207	0.2990	
hsa-miR-196b-5p	miR_196b_002215	002215	2.928	2.461	0.840	0.1938	Not expressed				
hsa-miR-197-3p	miR_197_000497	000497	0.009	0.201	22.211	0.3198	0.002	0.186	111.700	0.2083	
hsa-miR-199a-5p	miR_199a_000498	000498	0.523	0.201	0.385	0.1082	Not expressed				
hsa-miR-19a-3p	miR_19a_000395	000395	1.454	47.639	32.775	0.3903	1.911	161.150	84.342	0.1945	
hsa-miR-19b-1-5p	miR_19b_1__002425	002425	Not expressed				2.026	1.844	0.910	0.8747	
hsa-miR-19b-3p	miR_19b_000396	000396	1.307	2167.554	1658.030	0.2368	2.126	8235.957	3874.536	0.1808	
**hsa-miR-200a-3p**	**miR_200a_000502**	**000502**	1.393	847.936	608.576	0.2953	**2.087**	**2627.125**	**1258.981**	**0.0005**	*
hsa-miR-200a-5p	miR_200a__001011	001011	0.450	0.625	1.390	0.2569	0.022	0.186	8.265	0.1682	
hsa-miR-200b-3p	miR_200b_002251	002251	1.188	1440.145	1212.094	0.2830	1.484	3509.055	2364.104	0.2943	
**hsa-miR-200b-5p**	**miR_200b__002274**	**002274**	0.247	0.201	0.813	0.5291	**0.004**	**0.186**	**52.548**	**0.0104**	*****
hsa-miR-200c-3p	miR_200c_002300	002300	1.162	4256.241	3661.670	0.9614	1.396	13218.046	9466.432	0.1801	
hsa-miR-203a-3p	miR_203_000507	000507	1.466	9541.599	6510.098	0.2816	1.515	5975.552	3945.119	0.2809	
hsa-miR-204-5p	miR_204_000508	000508	1.004	860.416	857.321	0.6671	1.870	1018.986	545.049	0.1580	
hsa-miR-205-5p	miR_205_000509	000509	1.377	279.352	202.854	0.2912	1.146	170.191	148.533	0.2905	
hsa-miR-206	miR_206_000510	000510	1.724	5.190	3.011	0.3173	1.275	0.424	0.332	0.6351	
hsa-miR-20a-3p	miR_20a__002437	002437	1.241	5.937	4.784	0.5531	2.303	20.591	8.939	0.0998	
hsa-miR-20a-5p	miR_20a_000580	000580	1.218	1975.490	1622.172	0.3343	1.794	9384.614	5231.705	0.1148	
hsa-miR-20b-5p	miR_20b_001014	001014	0.892	17.076	19.142	0.9113	1.658	58.043	35.009	0.2521	
hsa-miR-210-3p	miR_210_000512	000512	1.573	725.034	461.053	0.2289	1.462	844.220	577.374	0.4252	
hsa-miR-212-3p	miR_212_000515	000515	1.628	6.071	3.730	0.5136	2.828	6.550	2.316	0.2213	
hsa-miR-214-3p	miR_214_002306	002306	0.383	0.201	0.525	0.4511	0.297	0.186	0.626	0.3096	
hsa-miR-215-5p	miR_215_000518	000518	33.442	806.392	24.113	0.0889	8.713	26.439	3.034	0.2277	
hsa-miR-21-5p	miR_21_000397	000397	1.100	753.293	684.983	0.1600	1.587	3033.257	1911.814	0.2477	
hsa-miR-216b-5p	miR_216b_002326	002326	0.712	0.201	0.283	0.3885	0.510	0.186	0.364	0.1644	
hsa-miR-218-5p	miR_218_000521	000521	1.381	68.840	49.842	0.2559	2.591	273.159	105.430	0.7204	
hsa-miR-219a-5p	miR_219_000522	000522	Not expressed				1.134	0.377	0.332	0.9850	
hsa-miR-221-3p	miR_221_000524	000524	1.631	1063.245	651.812	0.1415	1.099	917.486	834.778	0.0542	
hsa-miR-221-5p	miR_221__002096	002096	0.836	0.228	0.272	0.2858	0.705	0.271	0.385	0.4782	
hsa-miR-222-3p	miR_222_002276	002276	1.543	4891.653	3170.809	0.3905	1.042	5917.831	5678.521	0.6523	
hsa-miR-222-5p	miR_222__002097	002097	0.816	2.913	3.570	0.4604	0.066	0.186	2.814	0.0226	

**hsa-miR-223-3p**	**miR_223_002295**	**002295**	1.314	104.222	79.334	0.4134	**3.506**	**1274.378**	**363.441**	**0.0226**	*
hsa-miR-223-5p	miR_223__002098	002098	1.002	0.243	0.243	0.7745	0.977	0.324	0.332	0.2070	
hsa-miR-22-3p	miR_22_000398	000398	Not expressed				11.476	44.821	3.906	0.5415	
hsa-miR-224-5p	miR_224_002099	002099	1.276	56.546	44.300	0.4121	1.783	213.175	119.527	0.1004	
hsa-miR-22-5p	miR_22__002301	002301	1.125	6.927	6.159	0.2264	1.558	21.350	13.702	0.1985	
hsa-miR-23b-3p	miR_23b_000400	000400	Not expressed				1.834	185.129	100.919	0.5750	
hsa-miR-24-2-5p	miR_24_2__002441	002441	2.140	3.031	1.416	0.5026	0.423	0.684	1.617	0.1229	
hsa-miR-24-3p	miR_24_000402	000402	1.562	7284.808	4663.688	0.1699	1.517	9855.987	6496.326	0.2857	
hsa-miR-25-3p	miR_25_000403	000403	1.094	37.492	34.277	0.5854	1.765	144.208	81.712	0.0977	
hsa-miR-26a-1-3p	miR_26a_1__002443	002443	Not expressed				0.643	0.336	0.523	0.2108	
hsa-miR-26a-2-3p	miR_26a_2__002115	002115	Not expressed				0.506	0.186	0.367	0.1664	
hsa-miR-26a-5p	miR_26a_000405	000405	1.312	1710.230	1303.715	0.3353	1.800	5576.417	3098.006	0.1117	
hsa-miR-26b-3p	miR_26b__002444	002444	0.796	0.381	0.478	0.3287	1.273	1.291	1.014	0.0835	
hsa-miR-26b-5p	miR_26b_000407	000407	1.357	332.701	245.194	0.9085	1.993	976.434	489.825	0.8163	
hsa-miR-27a-3p	miR_27a_000408	000408	1.848	162.638	88.012	0.7168	1.975	167.996	85.041	0.0720	
hsa-miR-27a-5p	miR_27a__002445	002445	0.793	5.924	7.465	0.1255	0.330	0.496	1.502	0.0872	
hsa-miR-27b-3p	miR_27b_000409	000409	1.697	142.191	83.775	0.2927	1.609	412.070	256.061	0.9714	
hsa-miR-27b-5p	miR_27b__002174	002174	1.531	2.464	1.609	0.1476	1.231	8.275	6.722	0.1199	
hsa-miR-28-3p	miR_28_3p_002446	002446	1.101	103.720	94.218	0.8331	0.890	155.991	175.358	0.5505	
hsa-miR-28-5p	miR_28_000411	000411	1.655	44.071	26.630	0.2837	1.489	90.003	60.441	0.2537	
hsa-miR-296-5p	miR_296_000527	000527	2.603	14.527	5.581	0.7004	1.428	36.001	25.207	0.8342	
hsa-miR-29a-3p	miR_29a_002112	002112	1.263	28.484	22.548	0.1817	1.810	163.967	90.583	0.1270	
hsa-miR-29a-5p	miR_29a__002447	002447	2.124	3.356	1.580	0.6486	2.344	9.318	3.976	0.2995	
hsa-miR-29b-2-5p	miR_29b_2__002166	002166	Not expressed				1.496	1.043	0.697	0.6047	
**hsa-miR-29b-3p**	**miR_29b_000413**	**000413**	1.261	2.259	1.792	0.5727	**2.685**	**6.248**	**2.327**	**0.0241**	*
hsa-miR-29c-3p	miR_29c_000587	000587	0.891	55.344	62.130	0.2787	0.841	252.742	300.654	0.0885	
hsa-miR-29c-5p	miR_29c__001818	001818	1.544	0.563	0.364	0.5679	1.944	1.642	0.845	0.4091	
hsa-miR-301a-3p	miR_301_000528	000528	1.053	37.340	35.445	0.7005	1.864	168.470	90.374	0.1148	
hsa-miR-301b-3p	miR_301b_002392	002392	1.608	2.832	1.761	0.1688	1.108	2.793	2.520	0.4389	
hsa-miR-302a-3p	miR_302a_000529	000529	3.105	0.754	0.243	0.1829	Not expressed				

hsa-miR-302b-3p	miR_302b_000531	000531	Not expressed				0.976	0.324	0.332	0.9774
hsa-miR-30a-3p	miR_30a_3p_000416	000416	0.893	346.757	388.499	0.8584	1.485	721.226	485.575	0.0984
hsa-miR-30a-5p	miR_30a_5p_000417	000417	0.865	7338.502	8488.317	0.9139	1.733	70985.527	40958.146	0.1601
hsa-miR-30b-5p	miR_30b_000602	000602	0.893	8283.629	9280.758	0.4162	2.187	88024.468	40242.314	0.1379
hsa-miR-30c-5p	miR_30c_000419	000419	1.282	10506.707	8195.347	0.4264	2.155	50221.090	23309.686	0.1479
hsa-miR-30d-3p	miR_30d__002305	002305	0.972	5.643	5.808	0.9163	2.183	63.590	29.136	0.1923
hsa-miR-30d-5p	miR_30d_000420	000420	0.851	1845.687	2169.240	0.8786	1.833	18178.353	9919.361	0.0989
hsa-miR-30e-3p	miR_30e_3p_000422	000422	1.087	364.520	335.254	0.7358	1.224	656.011	536.019	0.0969
hsa-miR-31-3p	miR_31__002113	002113	1.515	36.874	24.338	0.3170	2.181	11.315	5.189	0.4930
hsa-miR-31-5p	miR_31_002279	002279	1.670	173.695	104.035	0.4459	1.224	58.782	48.013	0.1385
hsa-miR-320a	miR_320_002277	002277	1.322	1822.266	1378.846	0.2102	1.067	1713.194	1606.216	0.3278
hsa-miR-320b	miR_320B_002844	002844	1.086	12.556	11.561	0.8975	1.051	15.015	14.288	0.3506
hsa-miR-323a-3p	miR_323_3p_002227	002227	1.157	15.464	13.371	0.3069	0.813	0.793	0.976	0.2697
hsa-miR-324-3p	miR_324_3p_002161	002161	1.542	28.319	18.361	0.1603	1.566	70.454	44.995	0.1773
hsa-miR-324-5p	miR_324_5p_000539	000539	1.654	38.654	23.375	0.2375	1.894	77.216	40.760	0.4184
hsa-miR-32-5p	miR_32_002109	002109	1.236	0.624	0.504	0.3902	0.882	1.302	1.476	0.5704
hsa-miR-326	miR_326_000542	000542	Not expressed				1.015	1.326	1.306	0.1723
hsa-miR-328-3p	miR_328_000543	000543	1.437	187.473	130.486	0.8754	1.458	321.834	220.715	0.9691
hsa-miR-329-3p	miR_329_001101	001101	1.336	0.530	0.396	0.4778	Not expressed			
hsa-miR-330-3p	miR_330_000544	000544	0.651	0.484	0.743	0.6751	0.918	2.356	2.566	0.7387
hsa-miR-331-3p	miR_331_000545	000545	1.296	586.380	452.425	0.3410	1.426	1708.832	1198.110	0.3001
hsa-miR-331-5p	miR_331_5p_002233	002233	0.098	0.201	2.045	0.2990	0.939	0.822	0.875	0.4024
hsa-miR-335-3p	miR_335__002185	002185	Not expressed				2.526	4.808	1.903	0.0795
hsa-miR-335-5p	miR_335_000546	000546	1.581	108.953	68.898	0.2056	2.060	288.532	140.076	0.8144
hsa-miR-337-3p	miR_337_3p_002157	002157	0.990	1.279	1.292	0.9950	0.756	0.251	0.332	0.8178
hsa-miR-337-5p	miR_337_5p_002156	002156	2.525	3.058	1.211	0.5952	0.692	0.255	0.368	0.5111
hsa-miR-338-3p	miR_338_3p_002252	002252	1.222	1.565	1.281	0.7933	2.061	16.135	7.830	0.1909
hsa-miR-339-3p	miR_339_3p_002184	002184	1.223	6.254	5.115	0.5397	1.690	26.035	15.410	0.3305
hsa-miR-339-5p	miR_339_5p_002257	002257	0.985	28.772	29.221	0.9779	1.167	96.468	82.655	0.1884

hsa-miR-33a-3p	miR_33a__002136	002136	Not expressed				0.300	0.186	0.619	0.0616
hsa-miR-340-3p	miR_340__002259	002259	0.873	5.789	6.629	0.6361	1.544	19.392	12.559	0.1605
hsa-miR-340-5p	miR_340_002258	002258	1.137	3.578	3.147	0.5349	1.376	11.656	8.471	0.1155
hsa-miR-342-3p	miR_342_3p_002260	002260	1.552	130.270	83.924	0.9139	0.735	458.902	624.235	0.2600
hsa-miR-345-5p	miR_345_002186	002186	1.092	26.611	24.363	0.5057	1.317	104.084	79.023	0.3855
hsa-miR-34a-3p	miR_34a__002316	002316	0.857	10.142	11.830	0.2624	1.286	34.206	26.599	0.2866
hsa-miR-34a-5p	miR_34a_000426	000426	1.770	81.511	46.049	0.9184	1.389	144.092	103.754	0.4278
hsa-miR-34b-3p	miR_34b_002102	002102	0.773	1032.781	1336.612	0.7245	1.347	3012.594	2236.372	0.3592
hsa-miR-34b-5p	miR_34b_000427	000427	1.429	24.843	17.390	0.2050	1.696	117.556	69.318	0.3093
hsa-miR-34c-5p	miR_34c_000428	000428	1.344	87.997	65.471	0.3071	1.579	332.197	210.439	0.1765
hsa-miR-361-3p	miR_361_3p_002116	002116	Not expressed				0.399	0.186	0.465	0.2569
hsa-miR-361-5p	miR_361_000554	000554	1.378	30.553	22.179	0.2947	1.385	54.917	39.660	0.1695
hsa-miR-362-3p	miR_362_3p_002117	002117	1.681	0.933	0.555	0.3412	0.859	1.212	1.411	0.8360
hsa-miR-362-5p	miR_362_001273	001273	1.267	30.011	23.691	0.2856	1.209	30.927	25.575	0.3564
hsa-miR-363-3p	miR_363_001271	001271	1.025	2.398	2.339	0.5541	0.974	0.995	1.021	0.8826
hsa-miR-365a-3phsa-miR-365b-3p	miR_365_001020	001020	1.823	233.770	128.217	0.7878	1.259	69.976	55.585	0.3542
hsa-miR-367-3p	miR_367_000555	000555	44.662	68.034	1.523	0.1429	Not expressed			
hsa-miR-369-3p	miR_369_3p_000557	000557	3.060	1.696	0.554	0.1371	Not expressed			
hsa-miR-370-3p	miR_370_002275	002275	1.976	37.993	19.228	0.8832	Not expressed			
hsa-miR-372-3p	miR_372_000560	000560	1.219	0.431	0.354	0.7621	1.094	0.625	0.571	0.8010
hsa-miR-373-3p	miR_373_000561	000561	1.163	0.282	0.243	0.9559	1.553	0.516	0.332	0.2848
hsa-miR-374a-3p	miR_374a__002125	002125	Not expressed				1.350	0.608	0.450	0.7261
hsa-miR-374a-5p	miR_374_000563	000563	1.361	138.204	101.531	0.4201	2.624	454.589	173.262	0.0566
hsa-miR-374b-5p	miR_374_5p_001319	001319	2.059	102.310	49.681	0.1913	2.325	433.255	186.356	0.1694
hsa-miR-375	miR_375_000564	000564	1.544	413.395	267.678	0.4082	1.354	567.086	418.940	0.2453
hsa-miR-376a-3p	miR_376a_000565	000565	2.325	180.370	77.568	0.2599	1.736	6.600	3.802	0.1994
hsa-miR-376a-5p	miR_376a__002127	002127	2.040	0.495	0.243	0.0705	Not expressed			
hsa-miR-376b-3p	miR_376b_001102	001102	Not expressed				0.883	0.323	0.365	0.7463
hsa-miR-376c-3p	miR_376c_002122	002122	2.439	420.882	172.594	0.1139	1.266	8.022	6.339	0.6256
hsa-miR-378a-5p	miR_378_000567	000567	0.999	0.543	0.544	0.7148	0.645	0.271	0.420	0.3146

**hsa-miR-379-5p**	**miR_379_001138**	001138	**2.425**	**16.911**	**6.972**	**0.0378**	*	0.614	0.533	0.869	0.3493
hsa-miR-380-5p	miR_380_5p_000570	000570	0.963	0.390	0.405	0.7541		1.067	0.446	0.418	0.6934
hsa-miR-381-3p	miR_381_000571	000571	1.338	0.976	0.730	0.7666		1.505	0.624	0.414	0.4666
hsa-miR-382-5p	miR_382_000572	000572	1.327	74.993	56.524	0.6546		Not expressed			
hsa-miR-383-5p	miR_383_000573	000573	0.498	0.201	0.404	0.2472		0.123	0.186	1.508	0.0604
hsa-miR-409-3p	miR_409_3p_002332	002332	1.428	445.048	311.746	0.3814		0.858	7.583	8.837	0.5176
hsa-miR-409-5p	miR_409_5p_002331	002331	1.235	1.090	0.883	0.7112		Not expressed			
hsa-miR-410-3p	miR_410_001274	001274	1.910	113.805	59.586	0.1333		1.316	4.425	3.364	0.7401
hsa-miR-411-3p	miR_411__002238	002238	2.218	0.539	0.243	0.0880		Not expressed			
hsa-miR-411-5p	miR_411_001610	001610	2.190	51.949	23.726	0.1878		1.710	2.807	1.641	0.1777
hsa-miR-422a	miR_422a_002297	002297	1.065	0.310	0.291	0.9973		Not expressed			
hsa-miR-423-5p	miR_423_5p_002340	002340	0.950	3.553	3.738	0.6929		0.344	3.125	9.095	0.1387
hsa-miR-424-3p	miR_424__002309	002309	1.441	11.467	7.960	0.4459		0.252	0.186	0.735	0.6928
hsa-miR-424-5p	miR_424_000604	000604	1.408	1.593	1.131	0.1724		0.994	0.330	0.332	0.1167
hsa-miR-425-3p	miR_425__002302	002302	1.645	1.963	1.193	0.4584		1.376	3.462	2.515	0.2093
hsa-miR-425-5p	miR_425_5p_001516	001516	Not expressed					0.006	0.186	33.530	0.1395
hsa-miR-429	miR_429_001024	001024	1.143	102.851	89.958	0.4709		1.794	305.850	170.514	0.1331
hsa-miR-431-5p	miR_431_001979	001979	1.083	14.258	13.162	0.5866		Not expressed			
hsa-miR-432-5p	miR_432_001026	001026	1.971	5.951	3.019	0.8204		0.562	0.221	0.394	0.4306
hsa-miR-433-3p	miR_433_001028	001028	1.932	24.424	12.642	0.1723		Not expressed			
hsa-miR-449a	miR_449_001030	001030	0.957	146.620	153.131	0.3715		1.081	494.219	457.131	0.2395
hsa-miR-449b-5p	miR_449b_001608	001608	1.331	352.136	264.621	0.6987		1.217	1088.320	894.326	0.2157
hsa-miR-450a-5p	miR_450a_002303	002303	0.841	0.259	0.308	0.4709		Not expressed			
hsa-miR-451a	miR_451_001141	001141	1.089	6.798	6.242	0.8283		1.932	5.247	2.716	0.1513
hsa-miR-452-5p	miR_452_002329	002329	0.955	5.643	5.910	0.9165		1.796	43.378	24.149	0.2470
hsa-miR-454-3p	miR_454_002323	002323	1.101	43.046	39.109	0.4996		1.769	190.685	107.820	0.7874
hsa-miR-454-5p	miR_454__001996	001996	Not expressed					1.183	0.393	0.332	0.2824
hsa-miR-455-3p	miR_455_3p_002244	002244	0.913	19.112	20.934	0.4684		1.778	67.463	37.934	0.1093
hsa-miR-455-5p	miR_455_001280	001280	1.237	28.220	22.822	0.6344		1.843	86.627	47.000	0.2225
hsa-miR-483-3p	miR_483_3p_002339	002339	1.934	361.599	186.981	0.2066		0.017	0.186	10.832	0.0723

hsa-miR-483-5p	miR_483_5p_002338	002338	1.426	2295.200	1609.283	0.1659	0.640	87.766	137.142	0.7788	
hsa-miR-484	miR_484_001821	001821	0.864	975.266	1129.011	0.8248	1.625	2649.563	1630.298	0.2716	
hsa-miR-485-3p	miR_485_3p_001277	001277	1.351	7.970	5.897	0.7944	0.499	0.186	0.372	0.2180	
hsa-miR-487a-3p	miR_487a_001279	001279	0.684	0.257	0.376	0.4410	Not expressed				
hsa-miR-487b-3p	miR_487b_001285	001285	2.434	22.077	9.070	0.1159	0.601	0.450	0.749	0.5480	
hsa-miR-489-3p	miR_489_002358	002358	2.397	1.570	0.655	0.4750	0.946	0.612	0.647	0.7905	
hsa-miR-491-5p	miR_491_001630	001630	1.238	5.114	4.131	0.4887	1.389	14.421	10.382	0.3859	
hsa-miR-492	miR_492_001039	001039	1.109	0.269	0.243	0.9118	Not expressed				
hsa-miR-493-3p	miR_493_002364	002364	2.828	2.644	0.935	0.0791	Not expressed				
hsa-miR-494-3p	miR_494_002365	002365	1.776	13.272	7.472	0.1079	0.377	0.186	0.493	0.2289	
hsa-miR-495-3p	miR_495_001663	001663	2.395	99.878	41.699	0.1488	0.597	1.107	1.854	0.1657	
hsa-miR-499a-5p	miR_499_001352	001352	Not expressed				0.508	0.186	0.365	0.1691	
hsa-miR-500a-3p	miR_500_001046	001046	0.605	0.201	0.333	0.1930	0.372	0.186	0.499	0.1492	
hsa-miR-500a-5p	miR_500_002428	002428	1.125	12.480	11.096	0.7110	1.415	18.306	12.935	0.3434	
hsa-miR-501-3p	miR_501_3p_002435	002435	Not expressed				0.236	0.186	0.787	0.1800	
hsa-miR-501-5p	miR_501_001047	001047	0.211	0.201	0.954	0.2533	Not expressed				
hsa-miR-502-3p	miR_502_3p_002083	002083	1.302	3.597	2.763	0.5034	1.119	4.875	4.357	0.4716	
hsa-miR-502-5p	miR_502_001109	001109	1.489	4.450	2.990	0.4171	2.003	4.640	2.317	0.0624	
**hsa-miR-503-5p**	**miR_503_001048**	**001048**	1.883	2.982	1.584	0.1116	**2.129**	**1.002**	**0.471**	**0.0154**	*
hsa-miR-505-3p	miR_505_002089	002089	Not expressed				0.613	1.202	1.962	0.2553	
**hsa-miR-505-5p**	**miR_505__002087**	**002087**	1.287	0.392	0.305	0.9787	**0.191**	**0.186**	**0.971**	**0.0169**	*
hsa-miR-506-3p	miR_506_001050	001050	1.043	0.253	0.243	0.8285	Not expressed				
hsa-miR-508-3p	miR_508_001052	001052	1.206	2.800	2.322	0.8957	0.854	0.437	0.511	0.5969	
hsa-miR-509-5p	miR_509_5p_002235	002235	1.652	10.731	6.496	0.2161	1.017	0.989	0.972	0.7199	
hsa-miR-510-5p	miR_510_002241	002241	1.133	0.399	0.352	0.8506	Not expressed				
hsa-miR-511-5p	miR_511_001111	001111	Not expressed				0.875	0.564	0.644	0.6030	
hsa-miR-512-3p	miR_512_3p_001823	001823	0.399	0.589	1.474	0.3616	5.939	3.067	0.516	0.0854	
hsa-miR-520c-3p	miR_520c_3p_002400	002400	0.030	33.434	1104.389	0.7256	8.837	2.935	0.332	0.8373	
hsa-miR-520d-3p	miR_520D_3P_002743	002743	Not expressed				1.090	0.362	0.332	0.2737	
hsa-miR-520e	miR_520e_001119	001119	2.258	117.888	52.220	0.2950	1.387	122.332	88.169	0.1401	

hsa-miR-520f-3p	miR_520f_001120	001120	Not expressed				0.778	0.258	0.332	0.8075	
hsa-miR-526b-5p	miR_526b_002382	002382	0.484	0.201	0.416	0.1191	Not expressed				
hsa-miR-532-3p	miR_532_3p_002355	002355	1.317	90.471	68.690	0.3238	1.194	135.026	113.063	0.2650	
hsa-miR-532-5p	miR_532_001518	001518	1.293	232.150	179.485	0.3841	1.165	304.679	261.494	0.3963	
hsa-miR-539-5p	miR_539_001286	001286	1.576	33.561	21.293	0.3600	0.853	0.905	1.060	0.4026	
hsa-miR-542-3p	miR_542_3p_001284	001284	1.286	0.876	0.681	0.4621	Not expressed				
hsa-miR-543	miR_543_002376	002376	2.044	11.322	5.540	0.0884	0.596	0.320	0.538	0.3455	
hsa-miR-545-3p	miR_545_002267	002267	Not expressed				0.728	0.311	0.427	0.7356	
hsa-miR-548am-5phsa-miR-548c-5phsa-miR-548o-5p	miR_548c_5p_002429	002429	1.417	1.298	0.916	0.4741	1.609	1.595	0.991	0.0922	
hsa-miR-548b-5p	miR_548b_5p_002408	002408	1.757	1.415	0.805	0.5619	1.680	2.466	1.468	0.0334	
**hsa-miR-548d-5p**	**miR_548d_5p_002237**	**002237**	1.926	1.479	0.768	0.1540	**3.381**	**1.887**	**0.558**	**0.0148**	*****
hsa-miR-550a-5p	miR_550_002410	002410	1.122	0.273	0.243	0.9882	0.660	0.219	0.332	0.6238	
hsa-miR-551b-3p	miR_551b_001535	001535	3.954	330.768	83.653	0.5696	1.801	9.230	5.124	0.6488	
hsa-miR-551b-5p	miR_551b__002346	002346	1.797	0.436	0.243	0.5777	0.693	0.342	0.494	0.1498	
hsa-miR-564	miR_564_001531	001531	Not expressed				0.488	0.186	0.380	0.2437	
hsa-miR-572	miR_572_001614	001614	84.697	553.488	6.535	0.3676	0.185	8.173	44.275	0.7181	
hsa-miR-574-3p	miR_574_3p_002349	002349	1.109	775.409	699.173	0.4021	1.261	1027.929	814.976	0.1404	
hsa-miR-576-3p	miR_576_3p_002351	002351	1.535	0.373	0.243	0.1589	1.608	1.316	0.819	0.5226	
hsa-miR-577	miR_577_002675	002675	2.157	0.680	0.315	0.3232	0.845	1.826	2.162	0.9466	
hsa-miR-579-3p	miR_579_002398	002398	0.698	0.336	0.481	0.2369	1.315	1.154	0.878	0.7109	
hsa-miR-580-3p	miR_580_001621	001621	Not expressed				0.888	0.295	0.332	0.9990	
hsa-miR-589-3p	miR_589_001543	001543	Not expressed				0.217	0.186	0.855	0.0856	
hsa-miR-590-3p	miR_590_3P_002677	002677	1.356	1.108	0.817	0.2771	2.147	5.713	2.661	0.0867	
hsa-miR-590-5p	miR_590_5p_001984	001984	1.352	10.795	7.982	0.7665	2.099	41.233	19.647	0.1577	
hsa-miR-591	miR_591_001545	001545	1.555	0.378	0.243	0.6036	Not expressed				
hsa-miR-592	miR_592_001546	001546	0.970	0.236	0.243	0.8725	0.797	0.282	0.353	0.7239	
hsa-miR-595	miR_595_001987	001987	0.665	0.201	0.303	0.2375	Not expressed				
hsa-miR-597-5p	miR_597_001551	001551	1.915	1.234	0.644	0.3984	1.895	2.732	1.442	0.0272	
hsa-miR-598-3p	miR_598_001988	001988	1.171	4.930	4.210	0.4129	1.835	19.245	10.485	0.2359	

hsa-miR-601	miR_601_001558	001558	18.506	35.682	1.928	0.4125	0.540	1.807	3.346	0.8551
hsa-miR-604	miR_604_001567	001567	Not expressed				0.734	0.244	0.332	0.7803
hsa-miR-605-5p	miR_605_001568	001568	7.618	1.851	0.243	0.3405	Not expressed			
hsa-miR-616-3p	miR_616_002414	002414	Not expressed				1.763	0.694	0.394	0.6066
hsa-miR-616-5p	miR_616_001589	001589	1.062	0.258	0.243	0.8547	1.301	0.476	0.366	0.8965
hsa-miR-618	miR_618_001593	001593	11.032	2.680	0.243	0.4464	0.044	0.186	4.205	0.0702
hsa-miR-622	miR_622_001553	001553	1.108	0.842	0.760	0.8698	1.653	0.703	0.426	0.1886
hsa-miR-624-5p	miR_624_001557	001557	Not expressed				0.929	0.344	0.370	0.7802
hsa-miR-625-3p	miR_625__002432	002432	0.685	5.291	7.723	0.7131	0.674	13.657	20.256	0.0382
hsa-miR-625-5p	miR_625_002431	002431	0.986	0.289	0.293	0.3489	0.276	0.356	1.290	0.7269
hsa-miR-627-5p	miR_627_001560	001560	Not expressed				0.690	0.229	0.332	0.6923
hsa-miR-628-3p	miR_628_3p_002434	002434	1.782	0.433	0.243	0.2431	0.542	0.515	0.951	0.1502
hsa-miR-628-5p	miR_628_5p_002433	002433	0.609	0.486	0.797	0.4433	1.415	2.792	1.974	0.6872
hsa-miR-629-3p	miR_629_001562	001562	1.665	2.436	1.463	0.1528	0.915	4.004	4.375	0.8267
hsa-miR-635	miR_635_001578	001578	0.927	0.225	0.243	0.7128	Not expressed			
hsa-miR-636	miR_636_002088	002088	Not expressed				3.753	2.203	0.587	0.8788
hsa-miR-638	miR_638_001582	001582	0.114	0.201	1.761	0.2179	0.926	1.813	1.958	0.2341
hsa-miR-642a-5p	miR_642_001592	001592	1.058	18.506	17.492	0.8705	1.162	55.673	47.898	0.5363
hsa-miR-645	miR_645_001597	001597	0.954	0.608	0.638	0.6706	1.162	0.520	0.448	0.6887
hsa-miR-650	miR_650_001603	001603	0.321	0.201	0.627	0.2764	Not expressed			
hsa-miR-651-5p	miR_651_001604	001604	Not expressed				0.863	0.287	0.332	0.9712
hsa-miR-652-3p	miR_652_002352	002352	1.656	37.150	22.434	0.3443	1.273	85.442	67.106	0.3885
hsa-miR-654-3p	miR_654_3p_002239	002239	1.488	0.361	0.243	0.3559	Not expressed			
hsa-miR-656-3p	miR_656_001510	001510	5.208	2.930	0.563	0.2933	Not expressed			
hsa-miR-659-3p	miR_659_001514	001514	Not expressed				0.121	0.186	1.534	0.0607
hsa-miR-660-5p	miR_660_001515	001515	1.414	254.171	179.756	0.3550	1.523	370.275	243.126	0.1494
hsa-miR-664a-3p	miR_664_002897	002897	0.536	6.790	12.679	0.3054	1.592	43.902	27.580	0.6177
hsa-miR-671-3p	miR_671_3p_002322	002322	1.193	0.576	0.483	0.8681	1.670	1.559	0.933	0.7368
hsa-miR-708-5p	miR_708_002341	002341	1.280	10.431	8.149	0.5153	0.849	3.672	4.323	0.4958
hsa-miR-7-1-3p	miR_7__001338	001338	1.243	38.426	30.920	0.6591	1.812	85.496	47.194	0.8027
hsa-miR-744-3p	miR_744__002325	002325	0.845	0.651	0.771	0.7773	1.625	1.704	1.049	0.1976

hsa-miR-744-5p	miR_744_002324	002324	1.178	15.998	13.577	0.7086		1.061	37.625	35.457	0.0601
hsa-miR-758-3p	miR_758_001990	001990	1.888	1.359	0.720	0.5321		Not expressed			
hsa-miR-7-5p	miR_7_000268	000268	1.551	1.251	0.807	0.2507		0.500	0.742	1.484	0.1967
hsa-miR-765	miR_765_002643	002643	Not expressed					0.526	0.186	0.353	0.1872
hsa-miR-766-3p	miR_766_001986	001986	0.655	0.686	1.049	0.9383		0.044	0.186	4.185	0.4038
hsa-miR-769-5p	miR_769_5p_001998	001998	1.193	13.076	10.956	0.3245		1.305	29.248	22.410	0.0765
hsa-miR-885-5p	miR_885_5p_002296	002296	6.786	9.257	1.364	0.2505		1.572	0.673	0.428	0.2869
hsa-miR-888-5p	miR_888_002212	002212	0.206	2.727	13.269	0.6513		0.930	4.401	4.730	0.6371
**hsa-miR-889-3p**	**miR_889_002202**	002202	**2.094**	**6.021**	**2.876**	**0.0571**	*	0.659	0.242	0.368	0.5155
hsa-miR-890	miR_890_002209	002209	1.045	0.254	0.243	0.8868		1.533	0.548	0.357	0.6208
hsa-miR-891a-5p	miR_891a_002191	002191	0.793	0.522	0.658	0.7198		0.820	2.683	3.270	0.3763
hsa-miR-92a-3p	miR_92a_000431	000431	1.455	859.113	590.335	0.6657		1.294	2042.378	1577.911	0.1772
hsa-miR-93-3p	miR_93__002139	002139	1.085	6.134	5.652	0.6342		1.045	17.051	16.312	0.4800
hsa-miR-934	miR_934_002177	002177	Not expressed					0.641	0.318	0.496	0.3112
hsa-miR-93-5p	miR_93_001090	001090	1.114	119.883	107.607	0.0939		1.333	588.687	441.712	0.2640
hsa-miR-9-3p	miR_9__002231	002231	1.122	3.157	2.813	0.3800		0.435	0.186	0.427	0.1685
hsa-miR-942-5p	miR_942_002187	002187	1.151	3.127	2.716	0.4259		1.119	5.330	4.764	0.5080
hsa-miR-944	miR_944_002189	002189	0.740	0.201	0.272	0.5217		Not expressed			
hsa-miR-95-3p	miR_95_000433	000433	1.299	49.691	38.250	0.7692		1.343	107.858	80.327	0.0565
hsa-miR-9-5p	miR_9_000583	000583	0.943	22.283	23.625	0.6400		0.062	0.186	2.981	0.3138
hsa-miR-96-5p	miR_96_000186	000186	0.823	0.361	0.438	0.1710		1.416	0.667	0.471	0.1216
hsa-miR-98-5p	miR_98_000577	000577	13.054	10.585	0.811	0.3910		12.902	62.131	4.815	0.1585
hsa-miR-99a-3p	miR_99a__002141	002141	2.337	1.180	0.505	0.2049		2.283	6.068	2.659	0.2084
hsa-miR-99a-5p	miR_99a_000435	000435	1.520	979.404	644.395	0.3902		1.910	1252.246	655.584	0.3286
hsa-miR-99b-3p	miR_99b__002196	002196	1.509	2.079	1.378	0.6783		1.185	3.832	3.234	0.0214
hsa-miR-99b-5p	miR_99b_000436	000436	1.412	833.248	589.941	0.5279		1.312	1613.947	1230.417	0.2294
hsa-miR-1247°*	miR_1274A_002883	002883	1.262	14631.278	11589.847	0.3726		1.437	8289.140	5767.356	0.1656
hsa-miR-720*	miR_720_002895	002895	1.460	14041.066	9616.656	0.9790		1.107	7718.297	6973.357	0.1811
hsa-miR-378*	miR_378_002243	002243	3.935	5.074	1.289	0.4692		0.341	0.771	2.261	0.2189
hsa-miR-139-5p*	miR_139_5p_002289	002289	1.536	5.893	3.837	0.4913		1.622	7.958	4.905	0.4587
hsa-miR-1247B*	miR_1274B_00288	002884	1.240	326720.001	263545.684	0.0989		1.227	255409.342	208185.686	0.5448

	4									
hsa-miR-886-3p*	miR_886_3p_002194	002194	1.135	205.500	181.046	0.4159	0.711	76.172	107.124	0.6412
hsa-miR-1300*	miR_1300_002902	002902	Not expressed				0.784	0.400	0.510	0.7397
hsa-miR-1201*	miR_1201_002781	002781	Not expressed				0.722	0.240	0.332	0.7575
hsa-miR-886-5p*	miR_886_5p_002193	002193	0.978	203.710	208.300	0.5650	0.646	34.500	53.376	0.8679
hsa-miR-338-5p*	miR_338_5P_002658	002658	0.471	0.201	0.427	0.2030	1.919	6.546	3.410	0.9064

* miRNAs no longer present in the version of miRBase.
