# Supplementary material for: Extracellular Vesicles and Their miRNA Content in Amniotic and Tracheal Fluids of Fetuses with Severe Congenital Diaphragmatic Hernia Undergoing Fetal Intervention
Source: Cells. 2021 Jun 14;10(6):1493. doi: 10.3390/cells10061493 (PMC8231823; doi:10.3390/cells10061493)
Supplement: Supplementary file 1 [file cells-10-01493-s001.zip › cells-1255303 sm pdf/S4_table.pdf]

Table S4. miRNA validation in Amniotic and Tracheal Fluids

	miRNA	Mean copies/µl survivors	95% CI	Mean copies/µl non-survivors	95% CI	p-value
**Tracheal fluid**	**mir-223-3p**	203,9	119,5-348,1	526,8	260,2-1066,5	0,03
	**mir-503-5p**	11,6	8,2-16,4	19	13,4-26,8	0,05
	mir-548d-5p	78,6	53,2-116,1	128,3	18,0-211,0	0,13
	mir-29b-3p	290	185,8-452,7	424,5	258,3-697,7	0,26
	mir-17-3p	21,8	11,3-42,0	37,5	17,0-83,0	0,3
	mir-200a-5p	331,2	215,0-510,3	413	254,9-669,1	0,5
	mir-505-5p	47,2	29,1-76,5	37,8	21,9-65,1	0,55
	mir-200b-5p	561,1	336,2-936,3	643,5	363,0-1140,5	0,73
**Amniotic fluid**	**mir-889-3p**	103,9	66,2-162,9	209,7	134,1-327,7	0,03
	**mir-379-5p**	129,3	74,6-224,0	299,5	173,3-517,5	0,03
	mir-190-5p	137,3	85,3-220,9	240,9	150,0-386,7	0,1
